# Interactions between the helminth and intestinal microbiome in smallholder chicken farming systems

**DOI:** 10.3389/fvets.2023.1309151

**Published:** 2023-12-19

**Authors:** Mishumo Nemathaga, Rae M. Smith, Dikeledi P. Malatji

**Affiliations:** Department of Agriculture and Animal Health, College of Agriculture and Environmental Science, University of South Africa, Roodepoort, South Africa

**Keywords:** *Ascaridia galli*, chickens, helminth, intestinal microbiota, microbiota, nematodes

## Abstract

Helminth parasite infections are widespread in smallholder farming systems affecting farmers and livestock animals. There are pathogenic parasites that populate the gut of their host and coexist closely with the gut microbiota. The physical and immunological environment of the gut can be modified by parasites and microbiota creating a wide range of interactions. These interactions modify the development of infection, affects overall host health, and can modify the way a host interacts with its bacterial microbiota. In addition, where there is a high worm burden parasites will affect the health of the host and intestinal tract colonization. This review highlights key studies on the interaction between helminth parasites and the intestinal microbiome to understand the relationship between parasitic worm infections and gut microbiome health in chickens. Finally, the review discusses modulations, molecular changes, and the importance of helminth-microbiome interactions for the host.

## 1 Introduction

Poultry production plays an important role in extensive smallholder agriculture in developing countries as compared to cattle and goats, this highlights the importance of poultry husbandry in rural areas (1). A research study conducted from 2015 to 2020 by the World Bank presented stats on the proportion of livestock husbandry in 24 developing countries (2). The study was disaggregated by developing sub-regions and concluded that poultry was owned by 75, 78, and 57% of the sampled rural households in East Asia and the Pacific, Latin America and the Caribbean, and Sub-Saharan Africa, respectively (1, 2). This partially supports the importance of extensive poultry production within developing countries as a source of high-quality protein kept by most rural households within developing countries.

Extensive poultry production systems are free-range systems characterized by high mortality rates, open contaminated feeds, contaminated water sources, and litter systems within the surrounding environments, which allows for modes of parasites transmission from intermediate hosts such as ants, grasshoppers, earthworms, mites, and beetles (3). These intermediate hosts can withstand harsh environmental conditions and serve as food for chickens, making transmission of the infective stage of the parasites to chickens highly possible. In addition, microorganisms are also evident in eggs prior to hatching and are transferred from the mother via the chicken oviduct (4), or from the habitat through the pores in the eggshell (5). In addition, the gut microbiomes are also vulnerable to bacterial pathogens such as *Salmonella* and *Campylobacter* which can be a reservoir of antibiotic resistance and transmission leading to serious public health threats (6–8).

Tapeworms (cestodes), roundworms (nematodes), and flukes (trematodes) are helminth parasites that cause intestinal helminthiasis (9). With nematodes being the most economically important intestinal parasites in poultry (9). *Ascaridia galli* is one of the most common nematode parasite infecting chickens in different countries (10–15) with infections occurring more in chickens raised in backyard and free-range systems than in chickens raised in cage and poultry house production systems (16). According to a meta-analysis and systematic review study on the prevalence of helminth infections in chickens over time (based on articles published between 1942 and 2019), free-range and backyard systems had a significantly higher prevalence rate of helminth infections (84.8 and 82.6%) respectively than those reared in cage poultry production systems (63.6%) (16). Moreover, the pooled prevalence outcomes indicated that *A. galli* had the higher infection rate of 35.9% as compared to *Heterakis gallinarum* (28.5%), *Raillietina* spp. (19.0%), and *Capillaria* spp. (5.90%), with more than 30 helminth species identified. Furthermore, a study by Shifaw et al. (17) showed that chickens kept in floor production systems were infected with a minimum of one or more helminth parasite species.

The intestinal tract of a chicken is populated by a microbial population consisting of protozoa, fungi, bacteria, and viruses that have developed along with the host immune system (18–20). This microbial community has an important role in the physical performance, growth, and health of the host (21–24). In addition, the interaction between the host and intestinal microbiota aid in digestion of nutrients, immune system growth, and pathogen exclusion (25–28). Furthermore, a healthy microbial population is important for gut homeostasis and host metabolism, which affects animal physiology and health, with the host providing a tolerant environment and nutrients for bacterial colonization and growth (27).

The health and functionality of the chicken gut is influenced by the gastrointestinal microbiota and feed (19) with the gut microbiota forming protective barriers by adhering to the epithelial wall of the enterocyte to reduce the colonization of pathogenic bacteria (24). These bacteria also produce lower triglyceride and induce non-pathogenic immune responses and produce organic acids (e.g., lactic acid), fatty acids (acetic acid, butyric acid, and propionic acid) vitamins, (e.g., vitamin B group,) and antimicrobial compounds (e.g., bacteriocins) all of which offer nutrition and protection to the chicken (24, 29, 30).

## 2 Smallholder chicken production systems

**Figure 1:**
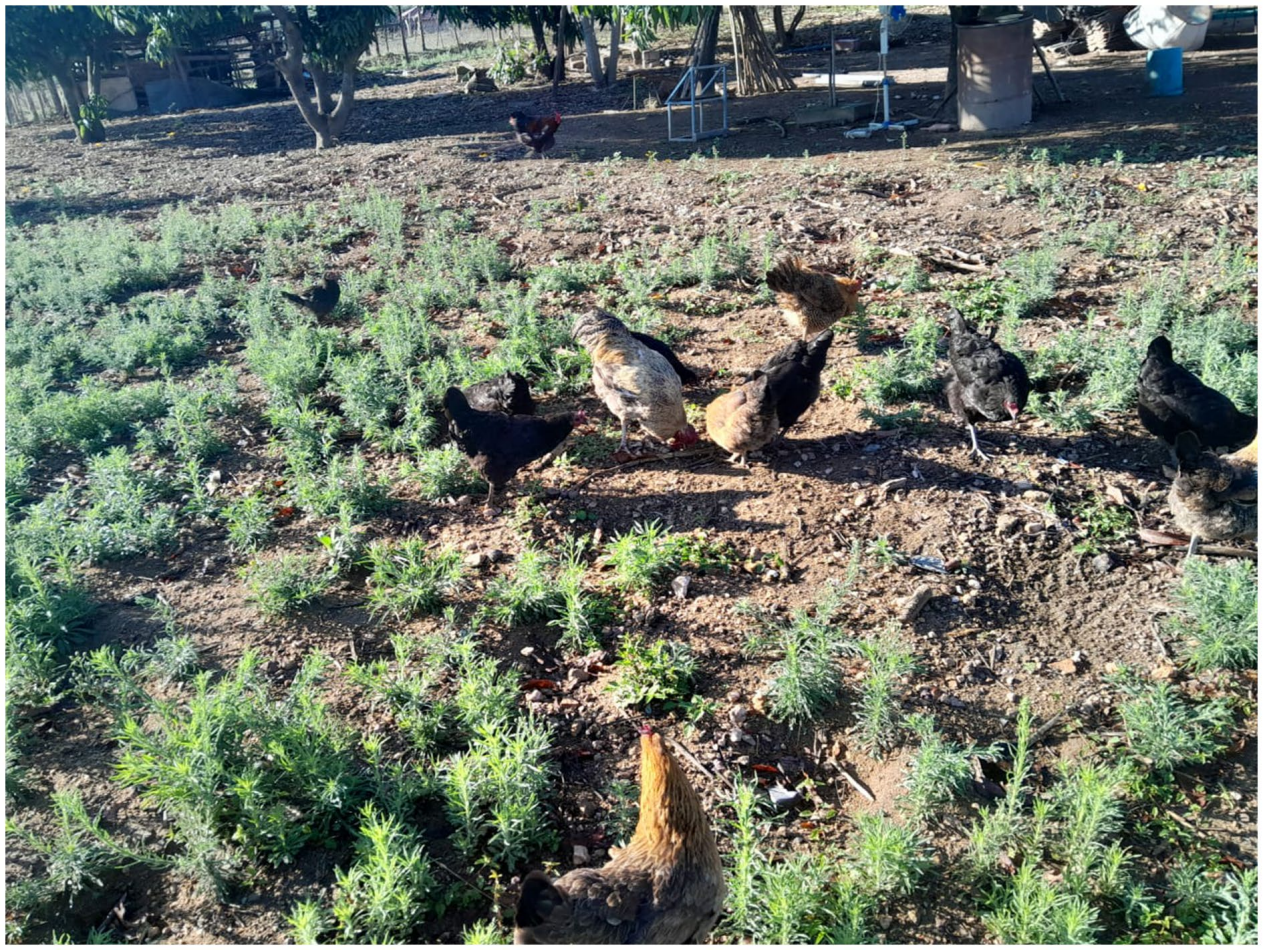
Indigenous chickens scavenging for feed to meet their nutritional needs.

Smallholder chicken production systems play a crucial role in poverty alleviation in resource-poor settings. The system tends to be characterized by chickens that get left to forage for themselves to meet their nutritional needs with little to no input (Figure 1) (31, 32) and is used particular by poor households, small-scale farmers, and landless communities located in rural settlements and villages (33). Chickens raised under such farming systems are susceptible to parasitic infections (34, 35) and encounter difficulties that include lack of quality feed ingredients, lack of quality water, lack of effective vaccination programs and unsuccessful marketing tactics, and mortality among others (36) due to not following well-managed and safe productive procedures and being free-range scavenging chickens. This approach creates a conducive environment that results in disease strain on scavenging chickens, because of various mixed ages in a flock and potential for disease transmission from other poultry species kept in the same area (37). Furthermore, no breeding programs exists in these systems and replacement stock is obtained from natural incubation (38).

## 3 Helminth parasites in chickens reared by smallholder farmers

Of the challenges experienced by chickens raised under a scavenging system, parasitism is a fundamental problem with both ectoparasites and endoparasites infecting chickens. Helminths are reported as one of the most common endoparasites worldwide found in scavenging chickens (16), with chickens raised under extensive smallholder farming systems being likely to pick up larvae, infective eggs, and intermediate hosts of parasites during scavenging (39). Furthermore, contact with infected feces causes a higher risk of endo-parasitism and *Salmonella* infection. A study conducted in selected villages of KwaZulu-Natal province of South Africa found *A. galli* to be a common endo-parasite present in all sampled sites with an infection rate of 58.3%, while *H. beramporia* and *Heterakis gallinarum* were present in only three sampled sites (14). The high pool prevalence of helminth infections is known to have an impact on welfare (40), production performance (41) of free-range chickens and susceptibility to secondary infections, thus causing significant economic losses for farmers (42).

Gastrointestinal helminth parasites are intricate multicellular organisms classified under various taxonomic families “but collectively share the capacity to downregulate the host immune response directed toward themselves (parasite-specific immunoregulation)” (43). A study by Malatji et al. (40) reported the downregulation of mast cell protease 1a-like, cytochrome P450 and apolipoprotein B in village chickens infected by *A. galli* parasites. These findings supported the results by Balqis et al. (44) that indicated the significant role played by mast cells in controlling *A. galli* infection. This is the strategy used by parasites to promote their survival by altering the activation of the immune response of the host during parasite infection. Furthermore, Malatji et al. (40) identified Arachidonic acid metabolism as one of the pathways that were significantly impacted in chickens infected by *A. galli* parasites. Arachidonic acid is able to regulate immune functions and Freeman et al. (45) showed that it can act as an inhibitor for Type 1 helper T cell (Th1) response.

These parasites are eukaryotic organisms that infect several hosts including livestock. Helminth infections are sub-clinical even when occurring in lower numbers but can suppress animal productivity and welfare through reduced weight gain and efficiency of feed utilization (46). In addition, factors such as the distribution of the intermediate hosts, their rate and the number of infective parasite eggs and larvae can influence helminth infection (47). Furthermore, studies have shown that the most widespread helminth parasites are *Heterakis gallinarum* and *A. galli* as reported in smallholder faming systems research (9, 13, 16, 48).

## 4 Chicken microbial community and mechanisms of gut helminth-microbiota interaction

While there is knowledge of the presence of helminth parasites in smallholder farming systems (16), there is little information on the interaction between helminth parasites and the gut microbiota. Several mechanisms are involved in the regulation of gut functionality and health, which makes it crucial to understand interactions between helminth parasites and the intestinal microbiome so that strategies are put together for the modulation of gut functionality and health to improve animal performance (49, 50).

Microbes are organisms that abundantly colonize the gastrointestinal tract of their host. These microbial organisms inhabit the intestinal tract of chickens, and are important for host metabolism and gut homeostasis, and affect the animal’s health and physiology (51). The gut microbiota is the microbial population that includes pathogenic, commensal and symbiotic microorganisms that are in and on multicellular organisms and are more abundant than germinal and somatic cells of the host (52). The collective genome of these symbionts is known as the microbiome (52). Thus, unraveling the development of the chicken microbiome and how it is influenced by different factors (35, 53, 54). These factors include dietary factors (such as dietary supplements, probiotics, and antibiotic growth promoters) that are of value, as the microbiota composition and functionality are linked with animal performance and health and can be modulated using dietary interventions (55, 56). Chickens host a range of microbial communities with the main sites carrying the most abundant microbiota being the skin, respiratory and reproductive tract, and gastrointestinal tract (20, 57, 58). The microbiota has a protective role in transmissible and non-transmissible diseases, and the gastrointestinal tract is the most vital site with the highest bacterial diversity and abundance (20). The intestinal tract helps maintain homeostasis, providing the organism with the ability to withstand physical, psychological, environmental stresses (49).

More than 900 bacterial species populate the gastrointestinal tract and aid in defense against pathogens, as well as digestion of food (35, 51). These bacterial species form a protective barrier through a variety of suggested mechanisms, including the production of antimicrobial factors, competition for nutrients and attachment to epithelial cells and preventing the opportunity for colonization of enteric pathogens (59, 60). Another distinctive trait of the gut microbiota is that commensal bacteria stimulate the early stages of the immunological system growth, including both the native and acquired immunologic responses, and regulate mucosal immunity (tolerance vs. inflammation), all depending on the microbial composition (19, 21, 61). Furthermore, the microbiota reduces and prevents colonization by exclusion, and the production of bactericidal substances and bacteriostatic (62). Also, the taxonomic composition of the microbiota is influenced by various components such as the animals age, diet, organ and animal antimicrobial usage (53). In addition, the intestinal microbiota is essential to hosts health, which makes it important to explain the mechanisms changing its diversity and composition.

Gastrointestinal helminths and bacteria populate the same ecological niche in livestock farming systems, where these microbes have relation with and influence each other (63). These intestinal helminths are host immune modulators that have developed spatially and temporally with the gut microbiota, which results in potential mechanistic interaction (64). Hence eukaryotic microbiota and parasites can modify the immunological and physical environment of the intestine which creates several chances of interaction. As a result, such interactions may modify infection results and have critical effect on the hosts health and diseases.

Interaction of bacteria with enteric helminths can contribute to the permanence of microbial prevalence. This can be seen in the link that exists between the gastrointestinal nematode (*Schistosoma* sp.) and bacterial *Salmonella* sp. Furthermore, *Schistosoma* sp. helps with the preservation of *Salmonella* sp. in the gastrointestinal tract of *Mus musculus*, due to bacteria connected to nematode folds (65). However, in chickens, enteritis per *Salmonella* sp. can reduce the presences of *A. galli* parasite established when the infection is late (66). This parasitic infection is susceptible to bacteria like *Pasteurella multocida* and *E. coli* (67, 68), however, *A. galli* is capable of producing bactericide molecules (69).

## 5 Modulation of intestinal communities by helminths

The chicken’s intestinal tract consists of the glandular stomach, known as the proventriculus, the gizzard known as the ventriculus and the small and large intestine (70, 71), with a metabolic function that develops the microbial community. It is densely colonized by a community of microbiota which include fungi, protozoa and bacteria that interact with the consumed feed and host. These intestinal helminths are prevalent in free-range and backyard poultry systems (47, 72) and represent a major constraint to poultry productivity.

Studies have shown that helminth infection can significantly alter the predicted metabolic potential and the composition of the gut microbiota (46, 73, 74), indicating that disturbance of the basal gut microbiota function may be a factor in reduced productivity in infected species (46). Additionally, infection can have a negative impact on animal health by increasing susceptibility to secondary infections and modulating host immunity (46).

## 6 Negative influence of bacteria on host health

Under normal conditions, bacteria tend to have unfavorable effect on gut health of the host as they produce some level of toxic compounds as a byproduct of metabolism when they compete with the host for nutrition (50). Village chickens carry different types of bacteria that affect their health (75). Studies have been conducted and detected *Salmonella* spp. in village chickens. Recently, studies reported the prevalence of 27 and 29% of *Salmonella* spp. on indigenous chickens from Tanzania and Iran, respectively (76, 77).

*Escherichia coli* is a motile Gram-negative bacterium belonging to the Enterobacteriaceae family. It is a natural inhabitant of the intestinal microbiota of chickens including their mucosal surface and it is present in the poultry habitat (26, 78). The majority of *E. coli* are non-pathogenic to the chicken host; however, 10–15% of *E. coli* isolated from the gastro-intestinal tract of broiler chickens may be infective (79). Avian Pathogenic *E. coli* (APEC) is a subset of extraintestinal pathogenic *E. coli* (ExPEC) that causes disease outside the animals’ gastrointestinal tract. It affects all poultry species in all types of production systems (80) and all chicken age groups (81). Furthermore, APEC result in localized and systemic infections in poultry, resulting in production loss and rapid mortality (82, 83).

*Enterococcus* spp. also showed to cause different diseases like femoral head necrosis, osteomyelitis, skeletal disease, and spondylitis. In addition, these bacteria are also linked to musculoskeletal disease in chickens (84, 85). The *Enterococcus* spp. is known to cause infections due to its intrinsic ruggedness (86).

## 7 The importance of helminth-microbiome interactions for the host

Over the course of evolution, livestock animals have coevolved with helminths and bacteria. The relationship between helminths microbiome and the host influences not only host-microbiome and host-helminth interactions but also the relationship between microbiome and helminth, with a significant impact on metabolic potential and host immunity (49, 87–89). There are benefits and costs to the host that are brought about by a normal gut microbial community (90, 91). The main benefits coming from the commensal microbiota are immune stimulation, contributions to host nutrition (feed conversion rates), competitive exclusion of non-indigenous microbes or pathogens (90).

The interactions between the GIT and microorganisms also influence the animals’ growth, stability of the microbial communities and animal’s health (92). Moreover, helminths and microbiota have immunomodulatory abilities and contribute to immune homeostasis within the host. Furthermore, gastrointestinal parasites can alter the diversity and composition of the intestinal microbiota in the host, with previous articles reporting that intestinal parasites can significantly change the composition and abundance of the intestinal microbiota (49, 50, 63, 93).

## 8 Biosecurity measures taken by rural/smallholder farmers to control helminth parasites

Helminth parasitic infection in smallholder farming systems is ubiquitous with negative impacts that invade livestock environments. These impacts require biosecurity measures that can benefit chicken production in smallholder farming systems. Biosecurity is a hygiene, segregation, or management procedure that aims at reducing any potential infectious microbes in and around farm environmental areas (94). To improve poultry production health monitoring and detection of parasitic infections is important, as this mitigates the spread of helminth parasites in smallholder farming areas, landless communities, and poor households. Control strategies are important in maximizing the possibilities of successful intervention which would assist in decreasing productivity losses, and range contamination for subsequent production cycles (42).

Biosecurity precautions are not essentially practiced across smallholder farmers in Africa as farmers in rural areas do not understand the potential risk to livestock and argue that the benefits of biosecurity measures do not outweigh the costs (95). Hence, they are faced with several challenges due to substandard biosecurity measures. These challenges can be seen in poultry raised through free-range systems with feed limited to what chickens can find on their own, which results in high prevalence rate of microbial infection, low productivity and high mortality rates (75). Biosecurity measures are an important part of any public health and animal plan as well as disease control and prevention for better survival and production output.

Although it may not be common in many smallholder farming systems, some of the biosecurity measures that are taken by some rural/smallholder farmers include isolation of livestock from sources of infectious contamination. This involves separating different livestock species from possible infectious areas to avoid exposing healthy livestock to contaminated areas (96). Farmers also limit the addition of new chickens into an existing flock, by keeping new poultry in isolation for 30 days before releasing them into an existing flock (96). This helps prevent cross-contamination which is also controlled by separating and identifying clean and unclean farming areas for decontamination and sanitization processes. Furthermore, privileged farmers control and minimize contamination by disinfecting work equipment and livestock areas, and using protective clothing for husbandry, which helps contribute to decrease and elimination of diseases (96). Additionally, other farmers provide clean supply of feed, water, litter and biocontainment throughout the farm as a biosecurity measures (97).

Furthermore, understanding the host–parasite relationship is important for the inception of strategic approaches to avoid increasing dependence on anthelmintics and frequent usage (98). There is also a paucity of research on the resistance of anthelmintic strategies (benzimidazoles) in parasites such as *A. galli*, indicating the importance of optimizing tools for the monitoring and detection of anthelmintic resistance in poultry parasites (99). The control of *A. galli* roundworms can also be carried out by simply breaking the reproductive cycle by eradicating the intermediate hosts such as earthworms (100). Moreover, it is important to improve management and hygiene among the flock before considering anti-parasite control, by frequently cleaning the environment in which chickens scavenge. Awareness strategies are also necessary to educate farmers on the *A. galli* parasite, the effects that helminths have on chickens, and how farmers can play a role in controlling infection among their chickens. Also, several ways have been implemented such as traditional, chemical, immunological, managemental, biological, and genetical strategies.

## 9 Conclusion

Poultry farming generates money for many smallholder farmers in developing countries through the sale of meat, eggs, manure, and live chickens. Poultry have coevolved with helminths and bacteria throughout evolution, with intestinal helminths and microbiota inhabiting the same ecological niche. The mode of relation can be indirect or direct and infection can have indirect impact on species health by increasing susceptibility to secondary diseases and modulation of the host immunity. Helminth infection influence intestinal microbiome and helminth-microbiota interaction indicates that microbiota reduces and prevents colonization. Therefore, it is imperative to understand the helminth-microbiota interaction to improve chicken productivity in low-output/low-input chicken production systems in order to develop effective control strategies against helminth infections in different parts of Africa and other developing countries.

## Author contributions

MN: Funding acquisition, Writing – original draft. RS: Writing – review & editing. DM: Conceptualization, Funding acquisition, Supervision, Writing – review & editing.
